# Mangiferin prevents myocardial infarction‐induced apoptosis and heart failure in mice by activating the Sirt1/FoxO3a pathway

**DOI:** 10.1111/jcmm.16329

**Published:** 2021-02-01

**Authors:** Lingli Chen, Santie Li, Jianyu Zhu, Anfu You, Xingzhou Huang, Xinchu Yi, Mei Xue

**Affiliations:** ^1^ Department of Neurology The First Affiliated Hospital of Wenzhou Medical University Wenzhou China; ^2^ School of Pharmaceutical Science Wenzhou Medical University Wenzhou China; ^3^ Department of Traumatology The First Affiliated Hospital of Wenzhou Medical University Wenzhou China; ^4^ Central Laboratory The First Affiliated Hospital of Wenzhou Medical University Wenzhou China; ^5^ People’s Hospital of Deyang City Deyang China

**Keywords:** apoptosis, FoxO3a, mangiferin, myocardial infarction, sirt1

## Abstract

Myocardial infarction (MI) commonly leads to cardiomyocyte apoptosis and heart failure. Mangiferin is a natural glucosylxanthone extracted from mango fruits and leaves, which has anti‐apoptotic and anti‐inflammatory properties in experimental cardiovascular diseases. In the present study, we investigated the role and detailed mechanism of mangiferin in MI. We used ligation of the left anterior descending coronary artery to establish an MI model in vivo, and cardiomyocyte‐specific Sirt1 knockout mice were used to identify the mechanism of mangiferin. For in vitro studies, oxygen and glucose deprivation (OGD) was used to mimic ischaemia in H9c2 cardiomyocytes. In mice, mangiferin treatment increased Sirt1 expression after MI, significantly reduced the infarct area, and prevented MI‐induced apoptosis and heart failure. Mangiferin reduced OGD‐induced cellular apoptosis in H9c2 cells. Meanwhile, Sirt1 knockout/silencing abolished the protective effects of mangiferin. Further studies revealed that mangiferin increased FoxO3a deacetylation by up‐regulating Sirt1, thus preventing apoptosis, and adenovirus‐mediated constitutive acetylation of FoxO3a restricted the anti‐apoptotic effects of mangiferin in vivo and in vitro. Our results indicate that mangiferin prevents cardiomyocyte apoptosis and the subsequent heart failure by activating the Sirt1/FoxO3a pathway in MI, and suggest that mangiferin may have an interesting potential in following studies towards clinical evaluation.

## 1 INTRODUCTION

Myocardial infarction (MI) is one of the most prevalent causes of morbidity and mortality worldwide.^1^, ^2^ Recent advanced methods such as stent technology, procedural techniques and adjunctive pharmacotherapy have been widely studied for the treatment of MI; however, the prognosis for patients with MI remains poor.^3^ The adult heart in mammals has poor regenerative potential,^4^ which means that the loss of cardiomyocytes cannot be restored after MI. Thus, preventing cardiomyocyte loss will be an efficient way to treat MI and promote recovery. Recent studies suggest that apoptosis, which is a form of programmed cell death, contributes to the progression of many diseases and leads to organ dysfunction.^5^ In MI, myocardial apoptosis is the main cause of cardiomyocyte loss and plays a critical role in the process of ventricular remodelling and subsequent heart failure.^6^, ^7^, ^8^ Thus, finding a safe and effective anti‐apoptotic therapeutic strategy for MI is important and will greatly improve the prognosis of patients.

Natural products are an important resource for the design and development of new drugs to target the cardiovascular system.^9^, ^10^ Among them, mangiferin (2‐β‐D‐glucopyranosyl‐1,3,6,7‐tetrahydroxy‐9H‐xanthen‐9‐one) is a novel bioactive compound derived from mango fruits and their by‐products.^11^ It is recognized as a safe natural product and has several beneficial effects, including anti‐oxidant, anti‐diabetic, anti‐inflammation, anti‐ageing and anti‐apoptotic actions.^11^, ^12^, ^13^ In the cardiovascular system, several studies have indicated that mangiferin has beneficial effects on cardiac inflammation, diabetic cardiomyopathy, coronary heart diseases and myocardial ischaemia‐reperfusion.^14^, ^15^ Recent studies also suggest that mangiferin has anti‐apoptotic activity in isoproterenol‐induced myocardial injury,^16^ but the effects and detailed mechanism of mangiferin in MI remain unclear.

Several studies have shown that mangiferin can modulate metabolism.^11^, ^12^, ^13^ As an important metabolism regulator, silent information regulator 1 (Sirt1) is a class III histone deacetylase that belongs to the sirtuin family (including Sirt1‐Sirt7).^17^ Sirt1 is a key regulator of tissue homoeostasis, and Sirt1 activation is commonly beneficial in many metabolic‐related diseases such as hypertension, obesity and diabetes.^18^, ^19^ These beneficial effects are caused by deacetylation of downstream proteins by Sirt1; for example, Sirt1 can deacetylate p65 and reduce inflammation, and it can also deacetylate p53, thus promoting cell survival.^20^ The beneficial effects of mangiferin have a close relationship with Sirt1 activity; for example, mangiferin regulates hepatic lipid metabolism through activation of a Sirt1/AMPK/SREBP‐1c pathway, and the protective effects of mangiferin in retina ischaemic injury are also mediated through Sirt1 activity.^21^, ^22^

The FoxO subfamily of forkhead transcription factors is an important downstream target of Sirt1.^18^ Sirt1‐mediated acetylation/deacetylation of the FoxO subfamily greatly influences its biological function.^19^ Among the FoxO subfamily, FoxO3a has a pivotal role in cell apoptosis, proliferation, DNA damage and tumorigenesis.^23^ FoxO3a is widely implicated in many types of diseases, including MI, and a transcriptional target of FoxO3a, the pro‐apoptotic protein Bim, is a key inducer of cardiomyocyte apoptosis in MI.^24^, ^25^ Interestingly, the deacetylation of FoxO3a, which can be mediated by Sirt1, down‐regulates the transcription of Bim, thus preventing apoptosis.^26^

In this study, we used ligation of the left anterior descending (LAD) coronary artery to construct an MI model in vivo, and inducible cardiomyocyte‐specific Sirt1 knockout (*Sirt1*‐iKO) mice were used to measure the protective effects of mangiferin in MI. Using these animal models along with in vitro experiments, we found that mangiferin protected against MI‐induced cardiomyocyte apoptosis and heart failure by activating the Sirt1/FoxO3a pathway.

## 2 MATERIALS AND METHODS

### 2.1 Animals

*Sirt1* flox/flox mice and transgenic mice with tamoxifen‐inducible Cre gene driven by cardiomyocyte‐specific α‐myosin heavy chain promoter (αMHC‐CreERT2) were constructed as previously reported (all under C57BL/6 background),^27^, ^28^ and were used to generate Sirt1 inducible knockout (*Sirt1*‐iKO) mice. *Sirt1* flox/flox mice were crossed with αMHC‐CreERT2 mice, and then, offspring were genotyped by polymerase chain reaction using the genomic DNA isolated from the tail. *Sirt1*‐iKO mice were generated by intraperitoneal injection of tamoxifen (75 mg/kg/day for 5 days) into male *Sirt1* flox/flox; αMHC‐CreERT2 mice (8‐12 weeks old), *Sirt1* flox/flox mice with tamoxifen injection, were used as a control group. Mice were housed for 1 week after tamoxifen injection to ensure efficient gene knockout.

All wild‐type (C57BL/6) mice, *Sirt1* flox/flox mice, and *Sirt1*‐iKO mice were housed in temperature‐controlled, pathogen‐free facility with ad libitum access to food and water. Animals received human care according to the National Institutes of Health guide for the care and use of laboratory animals (NIH Publications No. 8023, revised 1978). Animal experimental procedures were approved by the Institutional Animal Care and Use Committee of Wenzhou Medical University. All in vivo experiments were performed with male mice at the age of 8‐12 weeks old, and each group contains at least six mice. Male mice were included in this study because female mice were not considered to be an appropriate model in our study of MI, as female mice have attenuated remodelling, faster healing, and better cardiac function compared to male mice post‐MI, and the cardiac rupture rate of females will also be attenuated after LAD ligation according to previous reports.^29^, ^30^ The sample size of our study was determined following the guide of other appropriate studies.^31^, ^32^

### 2.2 Infarction model, drug treatment, measurement of the infarct size and survival rate

LAD ligation was performed to construct the MI model. C57BL/6 mice, *Sirt1* flox/flox mice, and *Sirt1*‐iKO mice were anesthetized with 2% isoflurane inhalation, and body temperature was maintained at 37°C with a heating pad. Then, the chest was opened by a horizontal incision, and LAD was ligated using a 7‐0 nylon suture. Ischaemia was confirmed by ECG and blanching of the left ventricle. Sham‐operated mice underwent the same procedure without ligation. Subsequent studies were performed on animals after the MI model was established successfully.

Mangiferin (Sigma) suspended in dimethyl sulfoxide (0.5% in phosphate‐buffered saline solution) was given to mice by intraperitoneal injection at 40 mg/kg/day for 14 days after LAD ligation, and the infarct size was assessed morphologically and calculated as the area of myocardial necrosis as a percentage of the whole myocardium by Masson's trichrome staining. The dose of mangiferin in our study was based on previous research.^15^, ^33^

Kaplan‐Meier survival curves were used to illustrate the cumulative survival of mice after MI ^34^, ^35^; this type of analysis is widely used in various diseases to measure the fraction of subjects surviving for a certain amount of time after treatment.^36^

### 2.3 Echocardiography

At the end of infarction experiments, C57BL/6 mice, *Sirt1* flox/flox mice, and *Sirt1*‐iKO mice from each group were anesthetized with 2% isoflurane inhalation, and heart function was measured by using transthoracic M‐mode echocardiography with an animal ultrasound system (Vevo 770, FUJIFILM VisualSonics). Heart rate, left ventricular anterior wall diastolic thickness and left ventricular posterior wall diastolic thickness were recorded; left ventricular end‐diastolic diameter and left ventricular end‐systolic diameter were measured to calculate percentage fractional shortening and percentage ejection fraction. The ejection fraction, fractional shortening, left ventricular anterior wall diastolic thickness and left ventricular posterior wall diastolic thickness are important markers for the evaluation of heart function.^34^, ^35^

### 2.4 Cell culture, RNA interference, oxygen‐glucose deprivation (OGD) and drug treatment

H9c2 rat cardiomyocytes were purchased from ATCC and cultured in high‐glucose (4500 mg/L) Dulbecco's modified Eagle's medium (DMEM; Gibco) supplemented with 10% foetal bovine serum (Sigma) and 1% penicillin/streptomycin (Gibco) in an incubator containing 95% air and 5% CO_2_ at 37℃. All cells used in the subsequent experiments were in passages 5‐10.

For RNA interference, H9c2 cells were transfected with Sirt1 siRNA (Santa Cruz Biotechnology) or control scrambled siRNA (Santa Cruz Biotechnology) using Lipofectamine 2000 (Invitrogen) for 12 h in Opti‐MEM (Gibco), and then, medium was changed to full‐growth DMEM, and cells were cultured for 12 h prior to experiments.

H9c2 cells were subjected to OGD to mimic myocardial ischaemia in vitro. Briefly, the full‐growth medium was changed to serum‐free and glucose‐free DMEM, and then, cells were moved to an incubator containing 95% N_2_ and 5% CO_2_ at 37℃ for 3 h or the indicated time‐points. Control cells were kept in normal medium under normal conditions. Mangiferin was added to the medium of cells subjected to OGD at the dose of 20 μM, unless otherwise stated.

### 2.5 Cell viability assay and lactate dehydrogenase (LDH) secretion

H9c2 cardiomyocytes were seeded in 96‐well plates at 2 × 10^4^ cells/well and then subjected to OGD for 1, 2 or 3h. H9c2 cardiomyocytes seeded in 96‐well plates without OGD were used as a control and represented OGD at 0 h. Cell viability was measured using a 3‐(4,5‐dimethylthiazol 2‐yl)‐2,5‐(diphenyltetrazolium bromide) (MTT) assay kit (Abcam) at the indicated time‐points according to the manufacturer's protocol. Briefly, cells were washed three times with phosphate‐buffered saline and treated with MTT reagent dissolved in serum‐free DMEM at 37 ℃ for 3 h. Then, MTT solvent was added and incubated for 15 minutes, and absorbance at 590 nm was measured using a microplate reader.

Lactate dehydrogenase assay kit (Nanjing Jiancheng Bioengineering Institute) was used to assess LDH release, which is an indicator of cell injury. According to the manufacturer's protocol, cell culture medium was collected and mixed with LDH reaction buffer for 30 minutes at room temperature, and then, absorbance was measured at 490 nm using a microplate reader.

### 2.6 Terminal deoxynucleotidyl transferase dUTP nick end labelling (TUNEL) staining

TUNEL staining was performed as a measure of apoptosis using the DeadEnd fluorometric TUNEL system (Promega) according to the manufacturer's protocol. For in vivo experiments, paraffin‐embedded heart sections (5 µm) were fixed using 4% paraformaldehyde in phosphate‐buffered saline for 1 h at room temperature, permeabilized with 0.5% Triton X‐100 for 20 minutes, and then stained with TUNEL reagent containing fluorescein‐12‐dUTP to detect apoptotic cell nuclei and with 4',6‐diamidino‐2‐phenylindole (DAPI) to stain all cell nuclei. The sections were also co‐stained with α‐actinin (1:100; Cell Signaling Technology, #6487) and the associated Alexa Fluor 647‐conjugated secondary antibody (1:200; Abcam, #ab150075) to confirm that apoptosis occurs in cardiomyocytes. For in vitro experiments, H9c2 cardiomyocytes were fixed using 4% paraformaldehyde in phosphate‐buffered saline for 15 minutes at room temperature, permeabilized with 0.5% Triton X‐100 for 5 minutes, and then stained by TUNEL and DAPI for detection of apoptosis. All images were captured by using a Leica SP8 confocal microscopy with the following parameter settings: DAPI (λex 364 nm; λem 460 nm), TUNEL (λex 488 nm; λem 520 nm) and α‐actinin (λex 652 nm; λem 668 nm). The number of TUNEL‐positive nuclei was counted in at least three randomly selected fields in the infarcted area of each slide. TUNEL‐positive nuclei of cardiomyocytes and interstitial cells were distinguished on the basis of their location on sections stained with or without α‐actinin, TUNEL‐positive nuclei which were not located in the area of α‐actinin‐positive myofibers were taken to be apoptotic interstitial cell nuclei, and the percentage of interstitial cell apoptosis were calculated by measuring the ration of apoptotic interstitial cell nuclei to total cell nuclei.

### 2.7 Adeno virus transfection

To restrict the deacetylation activity of Sirt1 on FoxO3a, C57BL/6 mice and H9c2 cardiomyocytes were transfected with constitutively acetylated mutants of FoxO3a. The constitutively acetylated mutants of FoxO3a (K271/290Q) were created as previously described,^37^ and adenovirus vectors expressing human FoxO3a containing this sequence with the cytomegalovirus (CMV) promoter and glutathione S‐transferase (GST)‐Tag (Ad‐FoxO3a‐CA) were constructed. Adenovirus vectors only expressing GST (Ad‐Con) were used as a control.

For adenovirus transfection in vivo, C57BL/6 mice were anesthetized with 2% isoflurane inhalation, and then, the chest was opened through a midline sternotomy. Adenovirus vectors (1.2 × 10^10^ PFU/mL) were directly injected into the left ventricle of the mice with a 32‐gauge needle. Each mouse received a 4‐point injection in a total volume of 100 µL. One week after injection, mice were subjected to LAD ligation. For in vitro experiments, H9c2 cells were transfected with the viral vectors at a multiplicity of infection (MOI) value of 20.

### 2.8 Western blotting and antibodies

Proteins from fresh heart tissue or cells were extracted using radio immunoprecipitation assay lysis buffer (Thermo Fisher Scientific) containing protease and phosphatase inhibitor cocktail (Abcam). Protein concentrations were detected by a Pierce bicinchoninic acid (BCA) protein assay kit (Thermo Fisher Scientific). Protein samples were prepared by mixing equal amounts of protein lysates with 3 × loading buffer and heating at 95℃ for 5 minutes. Proteins were separated by sodium dodecyl sulphate‐polyacrylamide gel electrophoresis (SDS‐PAGE) and transferred to a polyvinylidene fluoride (PVDF) membrane (Bio‐Rad), and then were subjected to immunoblotting for the following: cleaved caspase 3 (1:1000; Cell Signaling Technology), caspase 3 (1:1000; Cell Signaling Technology, #9662), Bcl‐2 (1:1000; Abcam, #ab59348), Bax (1:1000; Abcam, #ab32503), GAPDH (1:3000; Cell Signaling Technology, #5174), Sirt1 (1:1000; Cell Signaling Technology, #8469), acetylated‐p65 (1:1000; Abcam, #ab19870), p65 (1:1000; Abcam, #ab16502), acetylated‐p53 (1:1000; Abcam, #ab183544), p53 (1:1000; Abcam, #ab26), GST (1:1000; Abcam, #ab111947) and Bim (1:1000; Abcam, #ab32158). The secondary antibodies used are as follows: goat anti‐rabbit IgG H&L (HRP) (1:10 000; Abcam, #ab6721) and goat antimouse IgG H&L (HRP) (1:10 000; Abcam, #ab205719). After incubation with electrochemiluminescence (ECL) reagent (Millipore), protein bands were visualized in Amersham Image 600 system (GE Healthcare Life Sciences).

### 2.9 Immunoprecipitation (IP)

An IP assay was used to explore the acetylation level of FoxO3a. Fresh mouse heart tissue or H9c2 cells were lysed with non‐denaturing Nonidet P‐40 (NP‐40) lysis buffer (Beyotime) supplemented with protease and phosphatase inhibitor cocktail (Abcam). An amount of 500 μg protein was immunoprecipitated with anti‐FoxO3a antibody and PureProteome protein A/G mix magnetic beads (Merck Millipore) overnight at 4°C. Beads coated with the immune‐precipitated protein complex were washed and denatured at 95°C for 10 minutes. The immunoprecipitated proteins were then subjected to SDS‐PAGE. The antibodies used were as follows: FoxO3a (1:50; Cell Signaling Technology, #12829) and acetylated lysine (1:1000; Cell Signaling Technology, #9441).

### 2.10 Statistical analysis

Data were analysed using GraphPad Prism 5.0, and results were expressed as mean ± standard error of the mean (SEM). Statistical tests for normality or equality of variances were not applied because of the small sample sizes. An unpaired Student's two‐tailed t test was used to compare two groups; for comparisons between more than two groups, one‐way analysis of variance (ANOVA) was used. For in vivo studies, each group contained at least six mice, and no animals were excluded from the analyses. For in vitro studies, experiments were repeated at least three times. Kaplan‐Meier curves were assessed using the log‐rank test. A value of *P* < .05 was considered statistically significant.

## 3 RESULTS

### 3.1 Mangiferin alleviates MI‐induced cardiomyocyte apoptosis and heart failure

**FIGURE 1:**
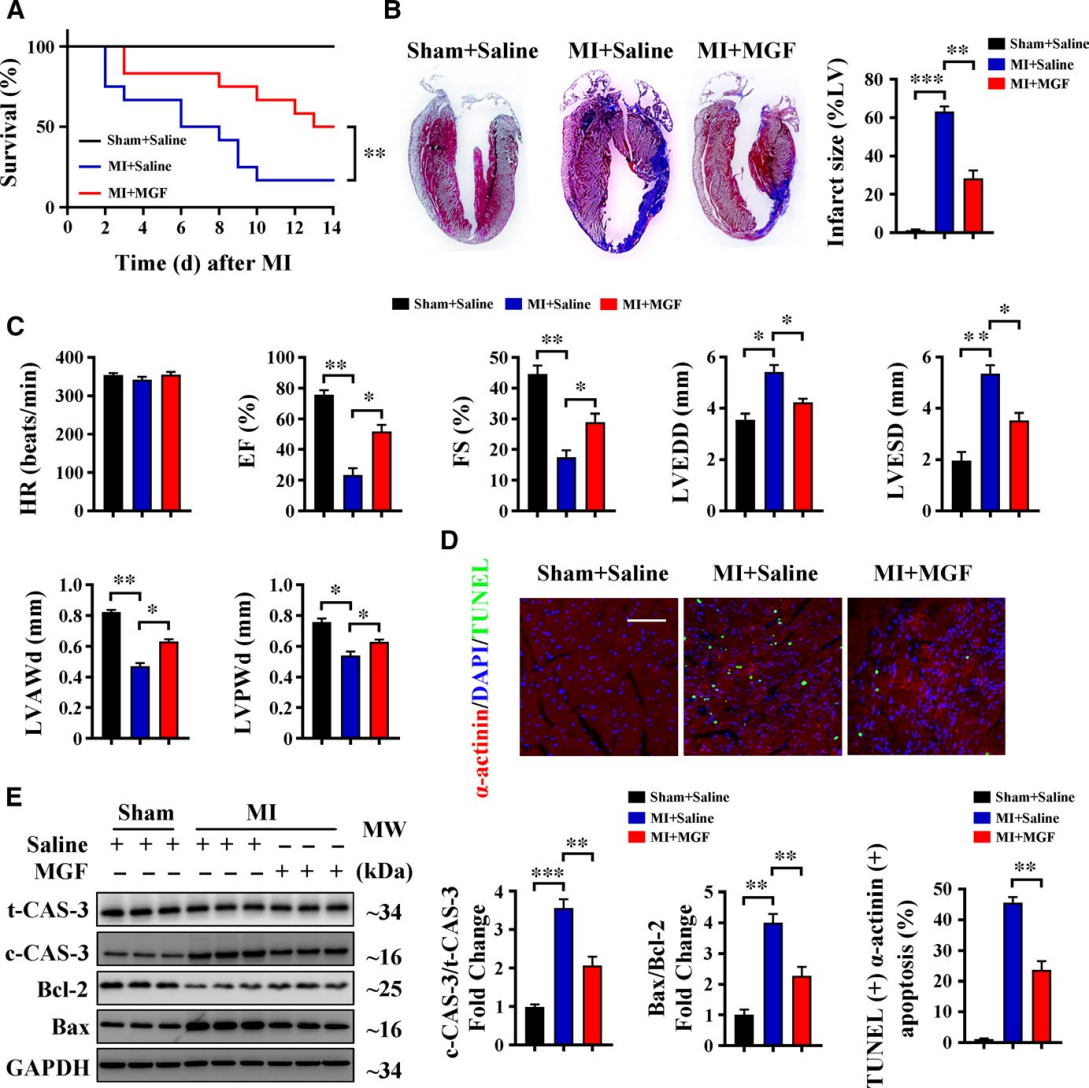
Mangiferin alleviates MI‐induced cardiomyocyte apoptosis and heart failure. (A‐E) C57BL/6 mice were subjected to sham or LAD ligation followed by the treatment with or without mangiferin for 14 d. A, Kaplan‐Meier survival curves of different groups of mice (n = 15 mice in each group). B, Representative images of Masson's trichrome staining of the heart sections and macroscopic measurements of the infarct size. C, M‐mode echocardiography data for HR, EF (%), FS (%), LVEDD, LVESD, LVAWd and LVPWd. D, Representative confocal scans for α‐actinin, TUNEL and DAPI staining (red, green and blue, respectively) of the heart sections and quantitative analysis (lower panel) of TUNEL + and α‐actinin + cells, Scale bar = 100 μm. E, Western blotting assay and quantitative analysis of cleaved caspase 3 expression and Bax/Bcl‐2 ratio in the heart tissue homogenates of different groups of mice. Unless otherwise stated, data are mean ± SEM for n = 6 mice in each group. *P < .05; **P < .01; ***P < .001. c‐CAS‐3, cleaved caspase 3; EF, ejection fraction; FS, fractional shortening; HR, heart rate; LVAWd, left ventricular anterior wall diastolic thickness; LVEDD, left ventricular end‐diastolic diameter; LVESD, left ventricular end‐systolic diameter; LVPWd, left ventricular posterior wall diastolic thickness; MGF, mangiferin; t‐CAS‐3, total caspase 3

MI was induced in wild‐type (C57BL/6) mice by LAD ligation, and then, mangiferin treatment was initiated by intraperitoneal injection for 14 constitutive days. Kaplan‐Meier survival analysis indicated that the post‐MI survival rate was significantly higher in the mangiferin‐treated group than in the saline‐treated group (Figure 1A), and Masson's trichrome staining showed that the infarct size was significantly smaller in mangiferin‐treated mice (Figure 1B). Our evaluation of heart function using echocardiography showed that mangiferin treatment had no effect on heart rate, but significantly increased the percentage of ejection fraction and fractional shortening after MI, and also significantly increased the left ventricular anterior wall diastolic thickness and left ventricular posterior wall diastolic thickness (Figure 1C). These results indicate that mangiferin alleviates MI‐induced heart failure. At the same time, TUNEL staining showed that cardiomyocyte apoptosis was lower in mangiferin‐treated mice (Figure 1D), and Western blotting showed that the level of cleaved caspase‐3 and the ratio of Bax/Bcl‐2, which are markers of apoptosis, were both decreased after mangiferin treatment (Figure 1E). Interestingly, from the TUNEL pictures we also observed several apoptotic interstitial cells; however, results indicated that mangiferin treatment could not attenuate interstitial apoptosis after MI (Figure S1A). These results indicate that mangiferin decreased cardiomyocyte apoptosis and improved heart function after MI.

### 3.2 Mangiferin alleviates OGD‐induced cell apoptosis and improves cell viability

**FIGURE 2:**
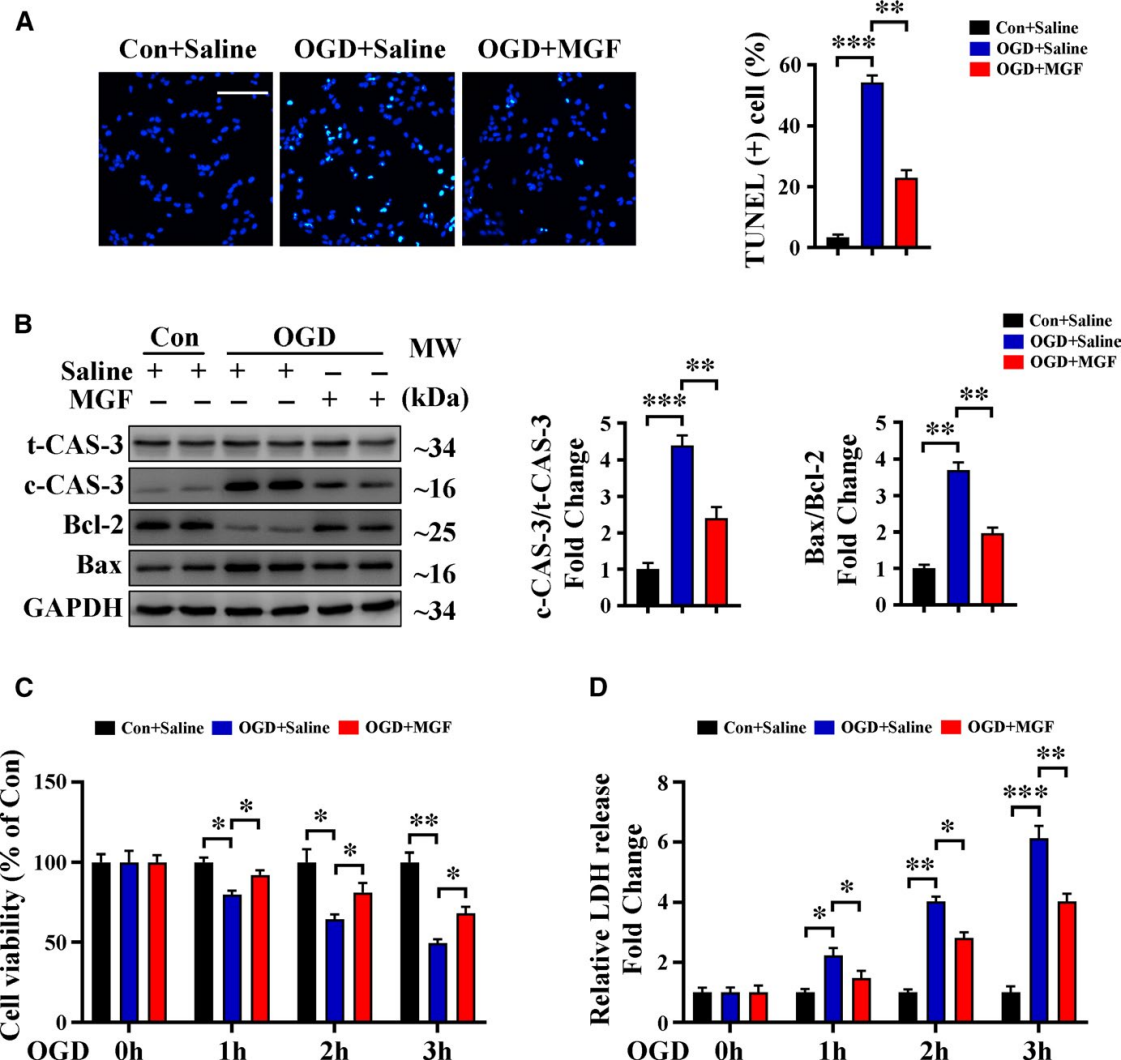
Mangiferin alleviates OGD‐induced cell apoptosis and improves cell viability. A‐D, H9c2 cardiomyocytes were subjected to control operation or OGD for 3 h with or without mangiferin treatment. A, Representative confocal scans for TUNEL and DAPI staining (green and blue, respectively) of the fixed cells and quantitative analysis of TUNEL + cells, Scale bar = 100 μm. B, Western blotting assay and quantitative analysis of cleaved caspase 3 expression and Bax/Bcl‐2 ratio in different groups of cells. C, MTT assay for the detection of cell viability in different groups of cells at indicated time‐points. D, LDH release assessment for the detection of cell injury in different groups of cells at indicated time‐points. Data are mean ± SEM for 3 independent experiments. *P < .05; **P < .01; ***P < .001. Con, control

To confirm our results showing that mangiferin decreased cardiomyocyte apoptosis, H9c2 cardiomyocytes were subjected to OGD to mimic myocardial ischaemia. TUNEL staining together with Western blotting of cleaved caspase‐3 and Bax/Bcl‐2 indicated that mangiferin reduced cardiomyocyte apoptosis in vitro (Figure 2A,B). Results of the MTT assay indicated that mangiferin treatment significantly improved cell viability after OGD (Figure 2C). To further investigate the protective effects of mangiferin in vitro, we performed an LDH assay as another indicator of cell injury. LDH release was significantly lower in H9c2 cells treated with mangiferin (20 μmol/L) than in saline‐treated cells after OGD (Figure 2D). We also treated H9c2 cells with varying concentrations of mangiferin (5, 10, 20, 40, 60 and 80 μmol/L); results of the MTT and LDH release assays indicated that 20 μmol/L of mangiferin produced better protective effects against OGD‐induced injury than other concentrations (Figure S1B,C). Furthermore, results of the MTT and LDH release assays also suggested that mangiferin alone had no detrimental effects on normal H9c2 cells (Figure S1D,E). Both of these experiments show that mangiferin treatment is an effective approach to alleviate cell apoptosis and improve cell viability after OGD.

### 3.3 Sirt1 is essential for the protective effects of mangiferin during MI

**FIGURE 3:**
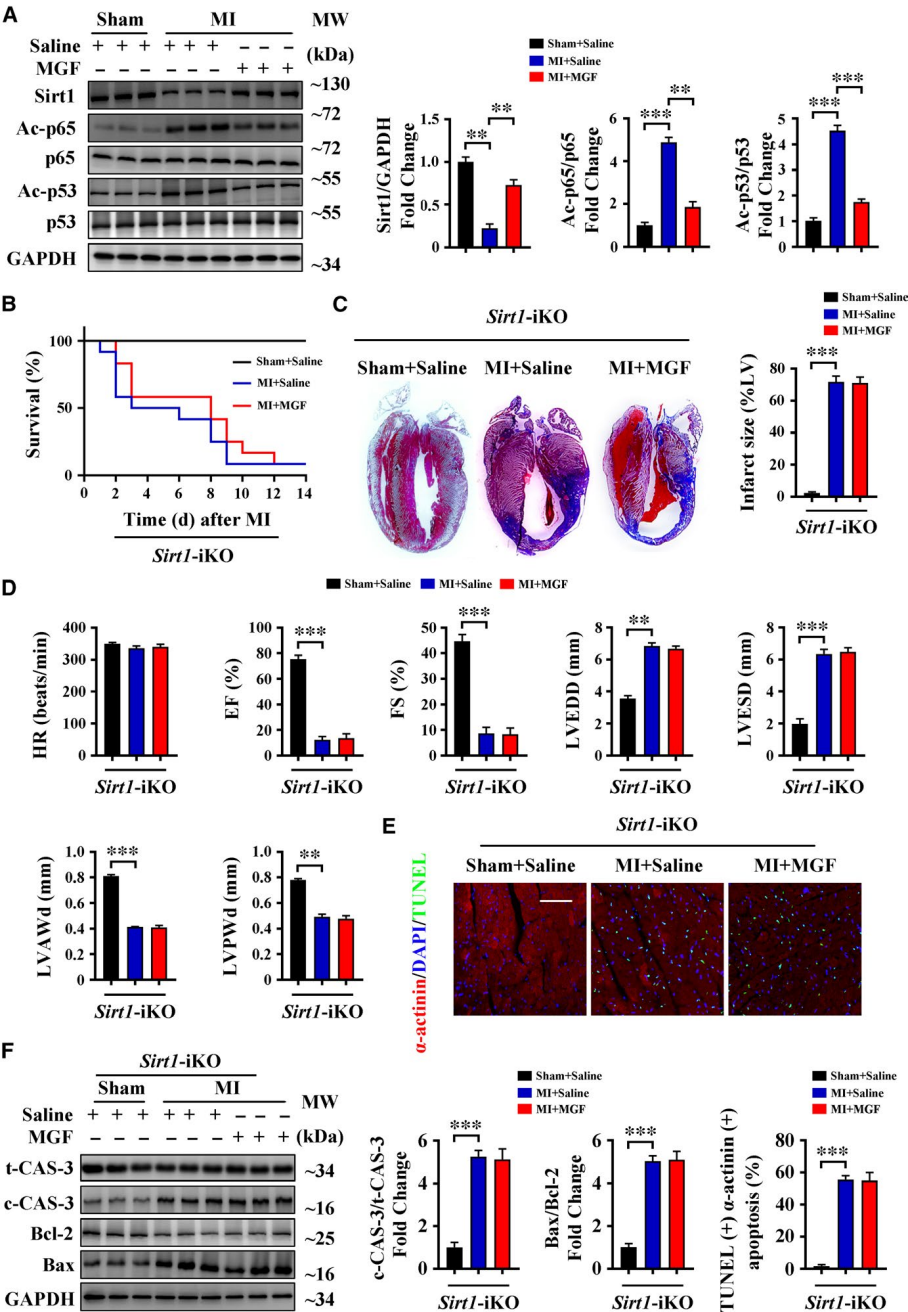
Sirt1 is essential for the protective effects of mangiferin during MI. A, C57BL/6 mice were subjected to sham or LAD ligation followed by the treatment with or without mangiferin for 14 d, Western blotting assay and quantitative analysis of Sirt1, acetylated p65 (Ac‐p65), p65, acetylated p53 (Ac‐p53) and p53 in the heart tissue homogenates of different groups of mice. B‐F, *Sirt1*‐iKO mice were subjected to sham or LAD ligation followed by the treatment with or without mangiferin for 14 d. B, Kaplan‐Meier survival curves of different groups of mice (n = 15 mice in each group). C, Representative images of Masson's trichrome staining of the heart sections and macroscopic measurements of the infarct size. D, M‐mode echocardiography data for HR, EF (%), FS (%), LVEDD, LVESD, LVAWd and LVPWd. E, Representative confocal scans for α‐actinin, TUNEL and DAPI staining (red, green and blue, respectively) of the heart sections and quantitative analysis (lower panel) of TUNEL + and α‐actinin + cells, Scale bar = 100 μm. F, Western blotting assay and quantitative analysis of cleaved caspase 3 expression and Bax/Bcl‐2 ratio in the heart tissue homogenates of different groups of mice. Unless otherwise stated, data are mean ± SEM for n = 6 mice in each group. *P < .05; **P < .01; ***P < .001

Several studies indicate that the effects of mangiferin are closely related to Sirt1 activation.^21^, ^22^ We also noticed that MI decreased Sirt1 expression in C57BL/6 mice and that mangiferin treatment significantly increased Sirt1 expression after MI, as well as the deacetylation of p65 and p53, both of which are downstream targets of Sirt1 (Figure 3A). This result indicates that mangiferin might protect the heart by up‐regulating the expression of Sirt1. To confirm this hypothesis, we generated *Sirt1*‐iKO mice (Figure S2A) and subjected them to LAD ligation followed by mangiferin treatment. Kaplan‐Meier survival analysis indicated that *Sirt1*‐iKO mice treated with saline and *Sirt1*‐iKO mice treated with mangiferin had the same survival rate (Figure 3B). Masson's trichrome staining together with echocardiography also showed that mangiferin had no protective effects in *Sirt1*‐iKO mice after MI, as indicated by the similar infarct size and heart function (Figure 3C,D). At the same time, TUNEL staining and Western blotting of cleaved caspase‐3 and Bax/Bcl‐2 showed that mangiferin did not prevent cardiomyocyte apoptosis in *Sirt1*‐iKO mice (Figure 3E,F), and interstitial apoptosis after MI was not changed in both of these animal models (Figure S1A). Together, these data indicate that mangiferin protects against MI by up‐regulating cardiomyocyte Sirt1 expression.

### 3.4 Sirt1 is essential for the protective effects of mangiferin during OGD

**FIGURE 4:**
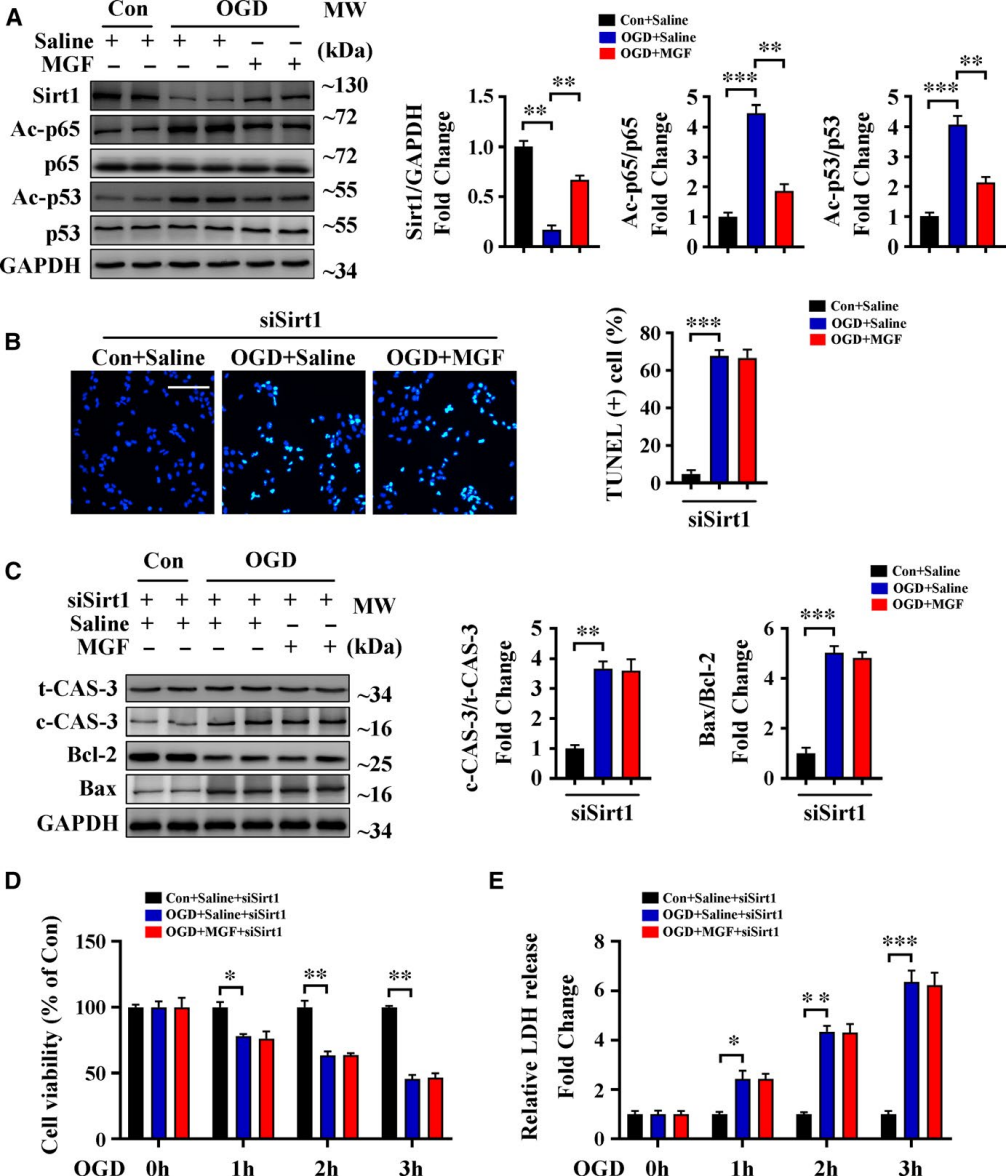
Sirt1 is essential for the protective effects of mangiferin during OGD. A, H9c2 cardiomyocytes were subjected to control operation or OGD for 3 h with or without mangiferin treatment, Western blotting assay and quantitative analysis of Sirt1, acetylated p65 (Ac‐p65), p65, acetylated p53 (Ac‐p53) and p53 in different groups of cells. (B‐E) H9c2 cardiomyocytes treated with Sirt1 siRNA were subjected to control operation or OGD for 3 h with or without mangiferin treatment. B, Representative confocal scans for TUNEL and DAPI staining (green and blue, respectively) of the fixed cells and quantitative analysis of TUNEL + cells, Scale bar = 100 μm. C, Western blotting assay and quantitative analysis of cleaved caspase 3 expression and Bax/Bcl‐2 ratio in different groups of cells. D, MTT assay for the detection of cell viability in different groups of cells at indicated time‐points. E, LDH release assessment for the detection of cell injury in different groups of cells at indicated time‐points. Data are mean ± SEM for 3 independent experiments. *P < .05; **P < .01; ***P < .001

We further validated an essential role for Sirt1 in the mechanism of action of mangiferin in H9c2 cardiomyocytes. Data indicated that mangiferin up‐regulated Sirt1 expression and caused deacetylation of p65 and p53 after OGD (Figure 4A); this result is consistent with our in vivo results. Silencing of Sirt1 expression using siRNA interference in H9c2 cells (Figure S2B) prevented the anti‐apoptotic effect of mangiferin after OGD; this was indicated by TUNEL staining and Western blotting of cleaved caspase‐3 and Bax/Bcl‐2 (Figure 4B,C). Similarly, in Sirt1‐silenced cells, mangiferin did not improve cell viability or prevent cell injury after OGD, as indicated by results from the MTT and LDH release assays (Figure 4D,E). Taken together, these results show that Sirt1 expression is essential for mangiferin to exert its protective effects after OGD.

### 3.5 Mangiferin protects the heart through Sirt1‐mediated deacetylation of FoxO3a during MI

**FIGURE 5:**
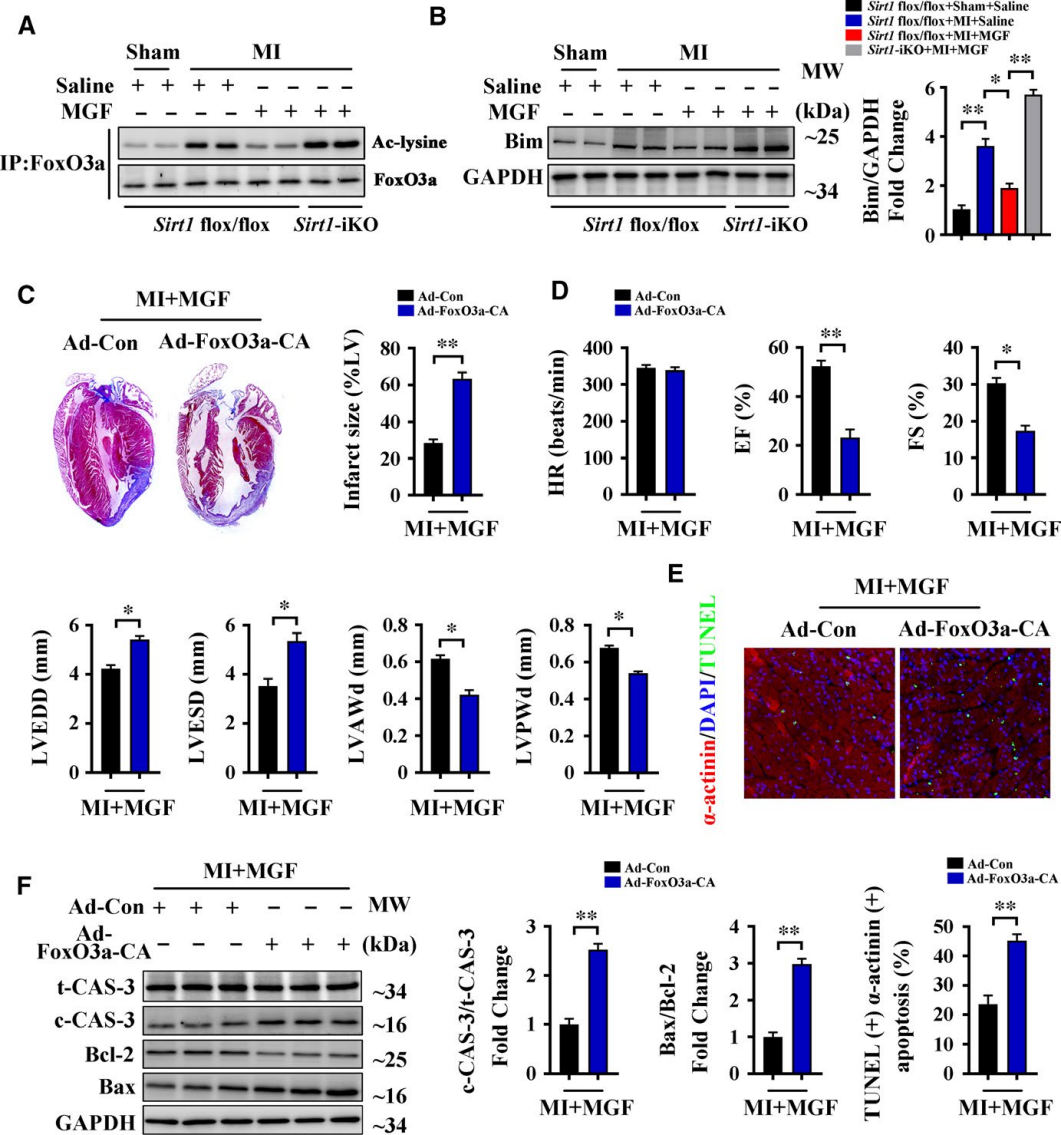
Mangiferin protects the heart through Sirt1‐mediated deacetylation of FoxO3a during MI. A‐B, *Sirt1* flox/flox and *Sirt1*‐iKO mice were subjected to sham or LAD ligation followed by the treatment with or without mangiferin for 14 d. A, IP assay showed the acetylation level of FoxO3a in the heart tissue homogenates of different groups of mice. B, Western blotting assay and quantitative analysis of Bim expression in the heart tissue homogenates of different groups of mice. (C‐F) C57BL/6 mice transfected with Ad‐Con or Ad‐FoxO3a‐CA vectors were subjected to LAD ligation followed by the treatment with mangiferin for 14 d. C, Representative images of Masson's trichrome staining of the heart sections and macroscopic measurements of the infarct size. D, M‐mode echocardiography data for HR, EF (%), FS (%), LVEDD, LVESD, LVAWd and LVPWd. E, Representative confocal scans for α‐actinin, TUNEL and DAPI staining (red, green and blue, respectively) of the heart sections and quantitative analysis (lower panel) of TUNEL + and α‐actinin + cells, Scale bar = 100 μm. F, Western blotting assay and quantitative analysis of cleaved caspase 3 expression and Bax/Bcl‐2 ratio in the heart tissue homogenates of different groups of mice. Data are mean ± SEM for n = 6 mice in each group. *P < .05; **P < .01; ***P < .001

The anti‐apoptotic effect of Sirt1 was mediated by deacetylation of several downstream targets in different organs or tissues.^38^ Our previous study demonstrated that FoxO3a, an important downstream target of Sirt1, plays an essential role in MI by regulating cardiomyocyte apoptosis.^39^ To explore the relationship between mangiferin and Sirt1/FoxO3a, we used an IP assay to detect acetylation of FoxO3a in our mouse models. Results from this experiment showed that mangiferin significantly reduced FoxO3a acetylation after MI in *Sirt1* flox/flox mice but not in *Sirt1*‐iKO mice. In *Sirt1*‐iKO mice, FoxO3a was still highly acetylated after mangiferin treatment (Figure 5A). At the same time, Western blotting indicated that the pro‐apoptotic protein Bim, which is known to be up‐regulated after FoxO3a acetylation,^26^, ^40^ was down‐regulated after mangiferin treatment in control mice but not in *Sirt1*‐iKO mice (Figure 5B). Together, these results show that mangiferin up‐regulates Sirt1 expression, which then causes deacetylation of FoxO3a in the infarcted heart.

To confirm our results, adenoviral vectors expressing constitutively acetylated FoxO3a (Ad‐FoxO3a‐CA) were constructed and injected into mouse myocardia (Figure S2C). We found that the protective effects of mangiferin in reducing infarct size and improving heart function were not seen in Ad‐FoxO3a‐CA‐transfected mice, as indicated by Masson's trichrome staining and echocardiography (Figure 5C,D). Furthermore, TUNEL staining and Western blotting of cleaved caspase‐3 and Bax/Bcl‐2 showed that mangiferin did not prevent cardiomyocyte apoptosis after MI when the mice were transfected with Ad‐FoxO3a‐CA vectors (Figure 5E,F), proving a necessary role for FoxO3a deacetylation in the anti‐apoptotic effect of mangiferin. At the same time, interstitial cell apoptosis was up‐regulated in Ad‐FoxO3a‐CA‐transfected mice after MI (Figure S1A), possibly because cardiac interstitial cells were also affected by the adenoviral vectors with the non‐specific CMV promoter. Taken together, these results indicate that mangiferin protects the heart by causing Sirt1‐mediated deacetylation of FoxO3a during MI.

### 3.6 Mangiferin alleviates OGD‐induced cell apoptosis and injury through Sirt1‐mediated deacetylation of FoxO3a

**FIGURE 6:**
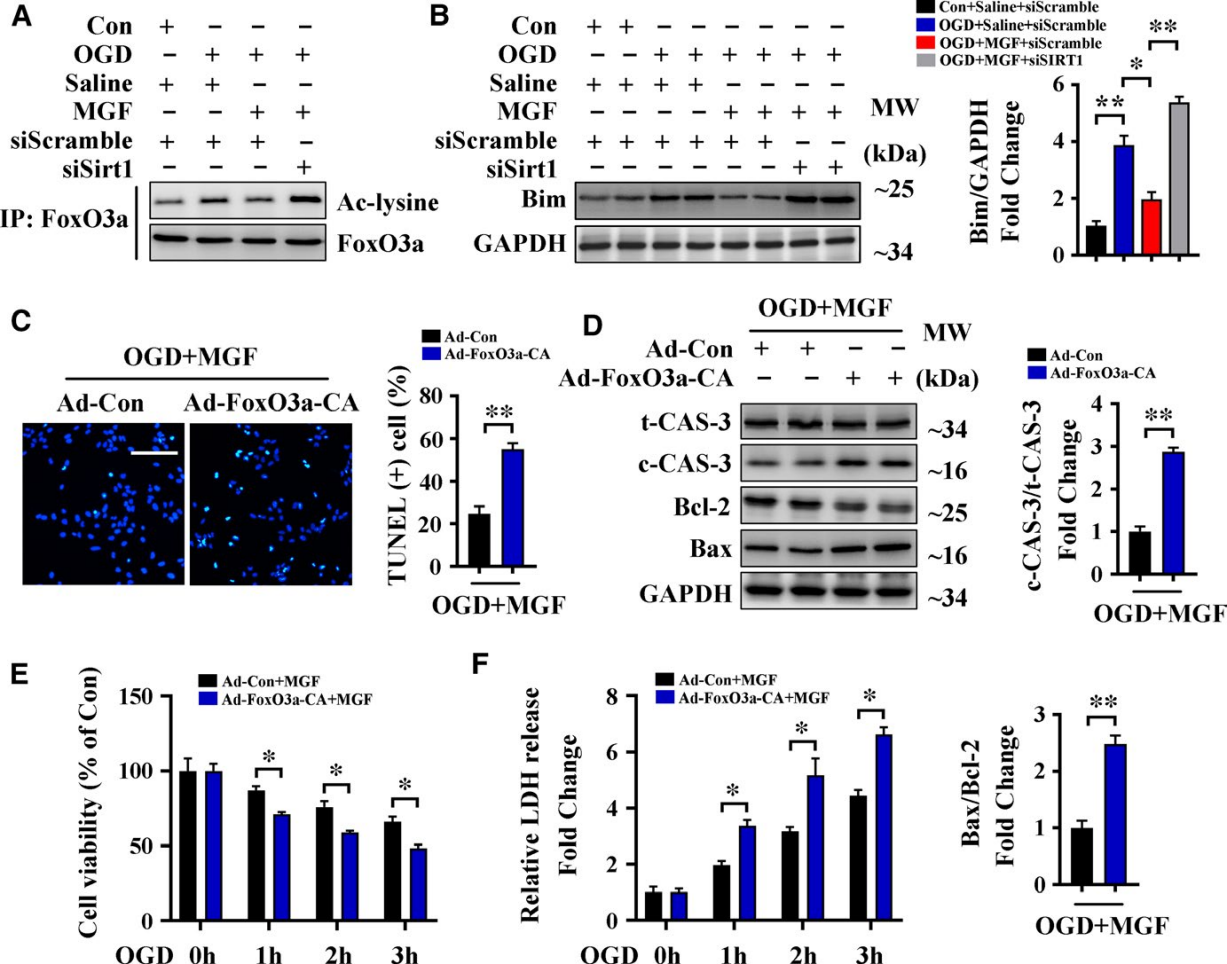
Mangiferin alleviates OGD‐induced cell apoptosis and injury through Sirt1‐mediated deacetylation of FoxO3a. A‐B, H9c2 cardiomyocytes treated with scramble siRNA or Sirt1 siRNA were subjected to control operation or OGD for 3 h with or without mangiferin treatment. A, IP assay showed the acetylation level of FoxO3a in different groups of cells. B, Western blotting assay and quantitative analysis of Bim expression in different groups of cells. C‐F, H9c2 cardiomyocytes transfected with Ad‐Con or Ad‐FoxO3a‐CA vectors were subjected to OGD for 3 h with mangiferin treatment. C, Representative confocal scans for TUNEL and DAPI staining (green and blue, respectively) of the fixed cells and quantitative analysis of TUNEL + cells, Scale bar = 100 μm. D, Western blotting assay and quantitative analysis (right and lower panel) of cleaved caspase 3 expression and Bax/Bcl‐2 ratio in different groups of cells. E, MTT assay for the detection of cell viability in different groups of cells at indicated time‐points. F, LDH release assessment for the detection of cell injury in different groups of cells at indicated time‐points. Data are mean ± SEM for 3 independent experiments. *P < .05; **P < .01; ***P < .001

We performed further in vitro experiments to confirm that mangiferin regulates cardiomyocyte apoptosis and improves cell viability through modulation of the Sirt1/FoxO3a pathway. Results from an IP assay indicated that OGD increased the acetylation of FoxO3a and that this effect was reversed by mangiferin treatment in H9c2 cells but not in Sirt1 siRNA‐transfected cells (Figure 6A). Similarly, mangiferin down‐regulated the expression of Bim after OGD in the presence of Sirt1 (Figure 4B); this result was consistent with results from our mouse models. Additionally, we transfected H9c2 cells with Ad‐FoxO3a‐CA vectors (Figure S2D), and TUNEL staining of these cells indicated that mangiferin did not prevent OGD‐induced apoptosis after transfection (Figure 6C). Western blotting of cleaved caspase‐3 and Bax/Bcl‐2 in Ad‐FoxO3a‐CA‐transfected cells treated with mangiferin showed a similar result (Figure 6D). Furthermore, results from MTT and of LDH release assays indicated that Ad‐FoxO3a‐CA transfection abrogated mangiferin‐mediated improvements in cell viability and prevention of cell injury after OGD (Figure 6E,F). Taken together, these data indicate that mangiferin alleviates OGD‐induced cell apoptosis and injury through Sirt1‐mediated deacetylation of FoxO3a.

## 4 DISCUSSION

In the present study, we demonstrated that mangiferin treatment is an effective approach to target apoptotic cell death in MI. Mangiferin significantly reduces cardiomyocyte apoptosis after LAD ligation or OGD, preserves cardiac function, and prevents the progression of heart failure after MI in mice. Previous studies researching the effects of mangiferin in cardiac injury have focused on the anti‐oxidative and anti‐inflammatory properties of mangiferin^14^, ^15^, ^16^; our study is the first to illustrate that Sirt1 is necessary for the protective effects of mangiferin in MI.

Sirt1 processes a wide variety of biological functions in multiple tissues and organs; in the heart, Sirt1 is considered to be related with cell apoptosis, cell survival, growth, senescence and metabolism.^17^, ^18^, ^19^, ^20^ Sirt1 is critical for heart embryo morphogenesis, knockout of Sirt1 in the embryonic stage will lead to a substantial rate of perinatal mortality associated with cardiac malformations, and interestingly, cardiac‐specific knockout of Sirt1 in adult mice shows no obvious phenotypes at basal conditions, but these mice will be more susceptible to cell death under stress conditions,^27^, ^41^, ^42^ indicating that Sirt1 is involved in the pathogenesis of cardiovascular diseases. Studies have indicated that Sirt1 activation is beneficial in doxorubicin‐, diabetes‐ and hypertension‐induced cardiomyopathy, as well as in MI.^17^, ^18^ In addition, Sirt1 agonists such as resveratrol are effective in the treatment of MI.^43^, ^44^ The results of our study, showing that mangiferin alleviates MI insult largely by up‐regulating cardiomyocyte Sirt1, contribute to the body of research showing that Sirt1 activation is beneficial in cardiovascular diseases. More importantly, by using cardiomyocyte‐specific Sirt1 knockout mice, we also indicate the essential role of Sirt1 in mangiferin‐mediated protective properties in MI; this helps us to better understand the effects of mangiferin in cardiac diseases.

Sirt1 deacetylates a large number of proteins, including FoxO transcription factors, which are the most highly regulated transcriptional mechanism involved in Sirt1 activation.^18^, ^19^, ^45^ Our results show that mangiferin treatment down‐regulates FoxO3a hyperacetylation, and this effect is absent after Sirt1 knockout or silencing, indicating that mangiferin regulates FoxO3a deacetylation through Sirt1 activation after MI. Of note, constitutive FoxO3a acetylation also abrogates the protective effects of mangiferin, indicating the presence of a Sirt1/FoxO3a regulating loop. Our results are in agreement with previous studies showing that FoxO3a‐dependent apoptosis plays a key role in cardiomyocyte apoptosis after MI^39^, ^46^; those studies showed that the acetylation of FoxO3a directly leads to the transcription of the pro‐apoptotic protein Bim, which induces apoptosis of cardiomyocytes. Our results indicate that Bim expression is down‐regulated after mangiferin treatment in the presence of Sirt1 in MI or after OGD and that constitutive acetylation of FoxO3a abrogates this effect of mangiferin. In addition, it should be noticed that other proteins or transcription factors can also be modulated by mangiferin, as both Sirt1 and FoxO3a have a large number of downstream targets.

Our results showed that mangiferin up‐regulated Sirt1 expression and promoted FoxO3a deacetylation during MI. Sirt1 is an epigenetic enzyme that induces epigenetic changes in its downstream targets to influence biological functions,^47^ which means that mangiferin can be regarded as an epigenetic bioactive compound. Several studies indicate that some functional food compounds have potential for the treatment of cardiac diseases through their action on the epigenome,^48^, ^49^ such as polyphenols, genistein and resveratrol; they can induce epigenetic activation of rescue genes or epigenetic inactivation of harmful genes, which help to promote cardiomyocyte survival after injury. As a bioactive compound derived from mango fruits, mangiferin could be used as a basis to design novel dietary approaches against ischaemic heart diseases, the action of which would be mediated through Sirt1 regulation of the epigenome.

The effect of mangiferin to prevent cardiomyocyte apoptosis means it may be used as an effective therapeutic strategy to target ischaemic heart diseases. Interestingly, our results showed that mangiferin did not have significant effects on interstitial cell apoptosis after MI. Cardiac interstitial cells contain a variety of cell types, such as leucocytes, macrophages, endothelial cells, smooth muscle cells, pericytes and fibroblasts.^50^, ^51^ Recent studies have suggested that cardiac interstitial cells are important players in organ injury response after MI, they represent regulatory functions in tissue homoeostasis and repair, and apoptosis of these cells also plays a respective role in the pathogenesis of MI.^50^, ^51^, ^52^ Our results demonstrated that mangiferin could not prevent cardiac interstitial cells from apoptosis in MI, which means that mangiferin protects the heart against MI mostly by preventing cardiomyocyte apoptosis.

In conclusion, we have shown that mangiferin has protective properties in the treatment of MI; mangiferin prevents cardiomyocyte loss by inhibiting apoptosis after MI, and thus prevents the development of heart failure. The effects of mangiferin are achieved by up‐regulation of Sirt1 expression and the related deacetylation of FoxO3a in cardiomyocytes. These data help us to better understand the role and detailed mechanism of mangiferin in MI, and suggest that mangiferin may have an interesting potential in following studies towards clinical evaluation.

## CONFLICT OF INTEREST

All authors declare that there are no conflicts of interest in this article.

## AUTHOR CONTRIBUTION

**Lingli Chen:** Conceptualization (lead); Data curation (equal); Formal analysis (equal); Investigation (equal); Methodology (equal); Resources (equal); Writing‐original draft (lead); Writing‐review & editing (equal). **Santie Li:** Conceptualization (lead); Data curation (equal); Formal analysis (equal); Investigation (equal); Methodology (equal); Resources (equal); Writing‐original draft (lead); Writing‐review & editing (equal). **Jianyu Zhu:** Conceptualization (equal); Data curation (equal); Formal analysis (equal); Investigation (equal); Methodology (equal); Writing‐review & editing (equal). **Anfu You:** Data curation (equal); Formal analysis (equal); Methodology (equal); Resources (equal). **Xingzhou Huang:** Data curation (equal); Formal analysis (equal); Methodology (equal); Resources (equal). **Xinchu Yi:** Data curation (equal); Formal analysis (equal); Methodology (equal); Resources (equal). **Mei Xue:** Conceptualization (lead); Data curation (equal); Formal analysis (equal); Funding acquisition (lead); Investigation (equal); Methodology (equal); Resources (equal); Supervision (lead); Writing‐review & editing (lead).

## Supporting information

Supplementary Material

## Data Availability

Please contact the corresponding author for data request.
