# Supplementary material for: Mangiferin prevents myocardial infarction‐induced apoptosis and heart failure in mice by activating the Sirt1/FoxO3a pathway
Source: J Cell Mol Med. 2021 Feb 1;25(6):2944–55. doi: 10.1111/jcmm.16329 (PMC7957271; doi:10.1111/jcmm.16329)
Supplement: Supplementary file 1 — Supplementary Material [file JCMM-25-2944-s001.docx]

**
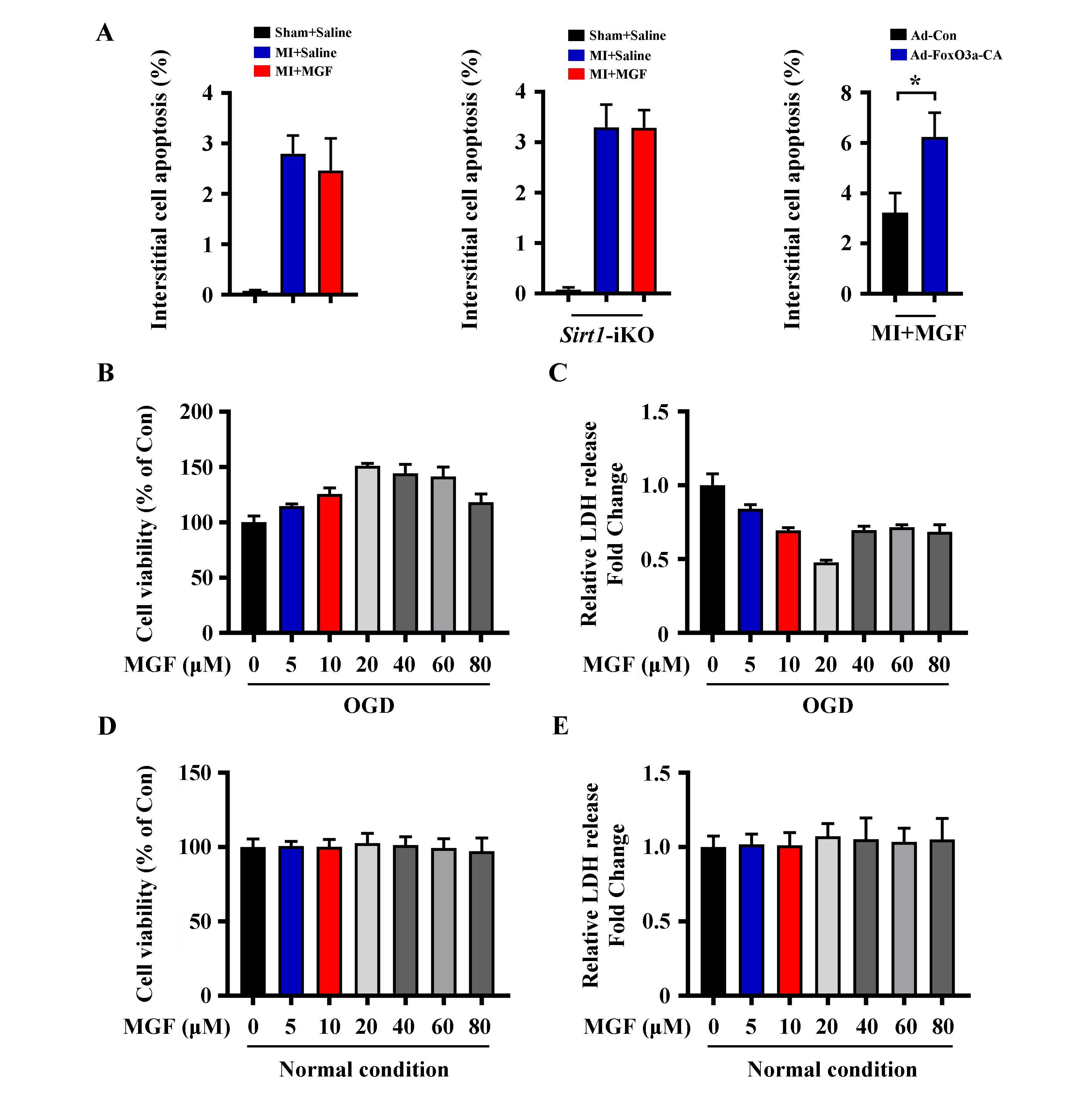
**

**Supplementary Figure 1****. Cardiac interstitial cell apoptosis in different group of mice and the effects of different doses of mangiferin on H9c2 cells during OGD and normal condition.** (A) Quantitative analysis of TUNEL+ cardiac interstitial cells in different group of mice. (B-C) H9c2 cardiomyocytes were subjected to OGD for 3 h. (B) MTT assay for the detection of cell viability in H9c2 cardiomyocytes treated with different doses of mangiferin after OGD. (C) LDH release assessment for the detection of cell injury in H9c2 cardiomyocytes treated with different doses of mangiferin after OGD. (D-E) H9c2 cardiomyocytes were treated with different doses of mangiferin for 24 h in normal condition. (D) MTT assay for the detection of cell viability in H9c2 cardiomyocytes treated with different doses of mangiferin in normal condition. (E) LDH release assessment for the detection of cell injury in H9c2 cardiomyocytes treated with different doses of mangiferin in normal condition. Data are mean ± SEM for n = 6 mice in each group or for 3 independent experiments. **P*<0.05.


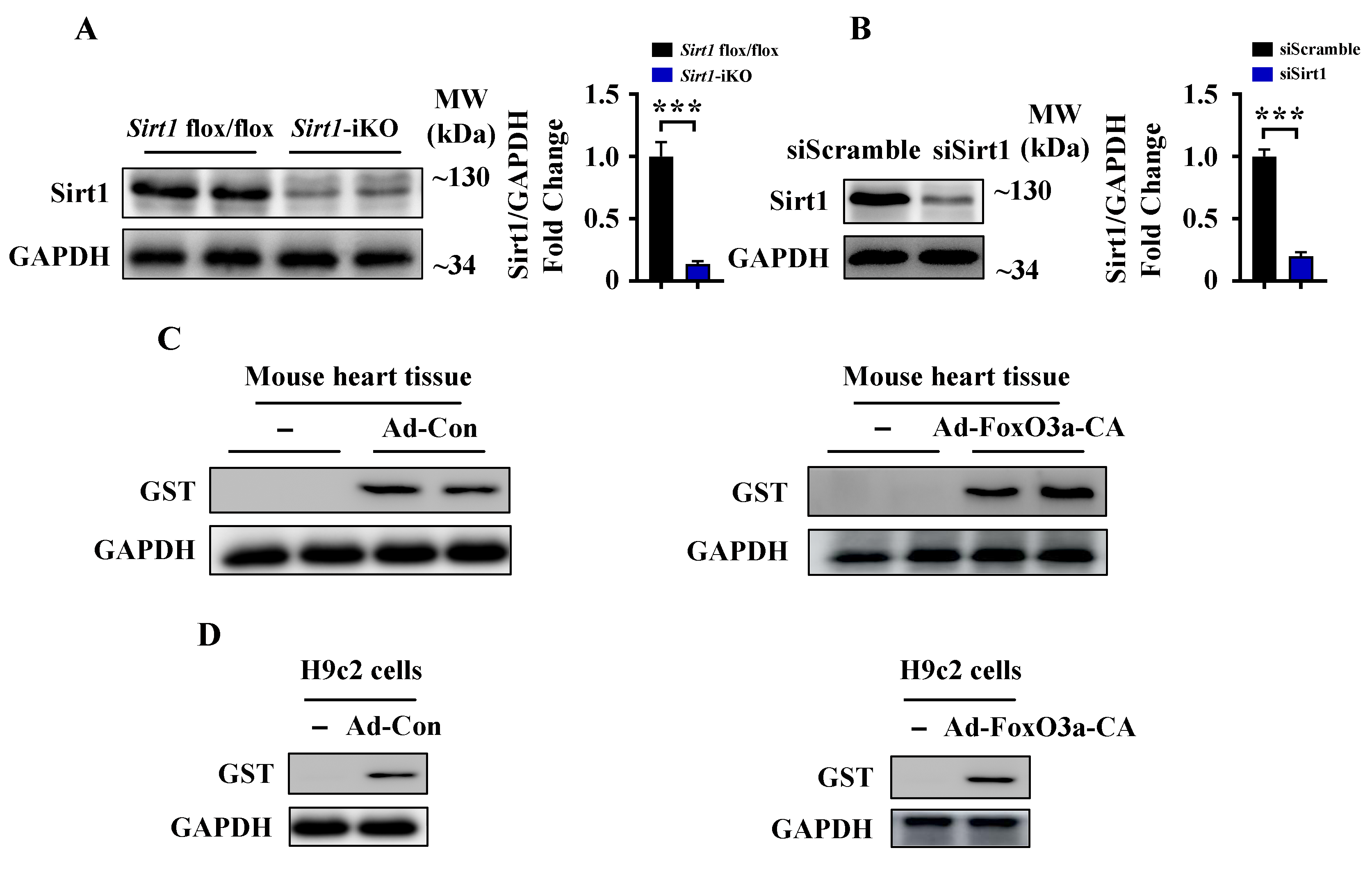


**Supplementary Figure 2. Identification of Sirt1 knockout/knockdown and adeno virus transfection both *in vivo* and *in vitro*.** (A) Western blotting assay and quantitative analysis of Sirt1 expression in the heart tissue homogenates of *Sirt1*-flox/flox mice or *Sirt1*-iKO mice. (B) Western blotting assay and quantitative analysis of Sirt1 expression in H9c2 cardiomyocytes treated with Scramble siRNA or Sirt1 siRNA. (C) Western blotting assay of GST expression in the heart tissue homogenates of non-transfected, Ad-Con or Ad-FoxO3a-CA-transfected mice. (D) Western blotting assay of GST expression in non-transfected, Ad-Con or Ad-FoxO3a-CA-transfected H9c2 cardiomyocytes. Data are mean ± SEM for n = 6 mice in each group or for 3 independent experiments. ****P*<0.001.
